# Editorial: Dialogues on preterm birth—causes and consequences, prevention and mitigation

**DOI:** 10.3389/fgwh.2023.1245570

**Published:** 2023-08-30

**Authors:** Vundli Ramokolo, Emmanuel Amabebe, Marta C. Cohen, Dilly O. C. Anumba

**Affiliations:** ^1^ HIV and Other Infectious Disease Research Unit, South African Medical Research Council, Cape Town, South Africa; ^2^ Department of Oncology and Metabolism, University of Sheffield, Sheffield, United Kingdom; ^3^ Histopathology Department, Sheffield Children's NHS FT, Sheffield, United Kingdom

**Keywords:** preterm birth (PTB), preterm delivery, newborn, causes, outcomes, prevention, management, perinatal

**Editorial on the Research Topic** Dialogues on preterm birth—causes and consequences, prevention and mitigation

Birth before 37 completed weeks gestation (Preterm birth, PTB), affects approximately 13.4 million newborns globally. The prevalence is even greater (∼35.3 million) when small and vulnerable newborns (SVN)—infants born small for gestational age and/or preterm—are considered. The global burden of PTB varies regionally and is disproportionately concentrated in sub-Saharan Africa and South Asia (1, 2). These regions also have the highest rates of infant mortality (3), as more than half of neonatal deaths are attributable to SVN (1). Target 3.2 of the sustainable development goals (SDG) was set and adopted by the United Nations in 2015 to reduce preventable neonatal and child deaths by 2030 (4). We are now midway through the tracking period and progress towards achieving this goal is slow and uneven globally. Furthermore, regions with the highest risk of neonatal and child mortality have the least amount of data available on SVN, which exacerbates inherent inequalities and impedes progress (1). Accurate, reliable, timely and more comprehensive data are required from all settings. Hence, this Research Topic was conceptualized to provide a medium for the dissemination of novel research on the estimation, etiology, prevention, and management of PTB; and the subsequent impact of PTB on the quality of life of affected mothers and communities. The Research Topic includes seven articles that address different aspects of PTB.

## Prevalence and risk factors

Given the paucity of data from sub-Saharan Africa, the article by Anto et al. gives very important insights into the prevalence of PTB in Ghana and the sociodemographic and clinical risk factors. Using a cross-sectional study approach, the authors collected data from pregnant women delivering in a tertiary hospital between May and June 2021. The study used skilled personnel to estimate gestational age by last menstrual period (LMP), clinical examination, and ultrasound. Socio-demographic and clinical data were obtained from the 209 study participants through interviews and data extraction from patient folders. The study reported that nearly 40% of neonates were preterm. The data also confirmed known risk factors for PTB such as preeclampsia and maternal obesity. The cross-sectional nature of the study is subject to temporality, therefore further research using more robust data collection approaches (such as longitudinal prospective cohort studies) is required to better understand risk factors for PTB and identify possible causal relationships in this context.

Risk factors of PTB are further explored by Musana et al. in 130 pregnant women recruited into a cohort study between April 2017 and February 2018 in a rural county in Kenya. The authors examine the hypothesis that maternal physiological stress (measured by cortisol and cortisone) during the second trimester may predict gestational length (based on LMP and ultrasound) better than psychological stress (measured by self-reported stress). They further hypothesize that both maternal obstetric risk [measured by obstetric medical risk index (OMRI)] and fetal sex would influence the relationship between psychological and physiological stress and gestational length. The data did not support these hypotheses. However, the findings did confirm known associations between PTB and obstetric risks such as prior preterm deliveries, maternal genitourinary tract infections and influenza.

## Mechanisms

The role of infections and inflammation on PTB risk is further discussed in a narrative review by Tantengco and Menon. This review summarizes evidence on (1) the epidemiology of both bacterial and viral cervical infections in pregnancy, (2) possible cellular mechanisms that link cervical infections and PTB through inflammation and subsequent premature cervical ripening, (3) endogenous cervical defense mechanisms against infection and inflammation, and (4) potential prognostic inflammatory biomarkers for premature cervical ripening and PTB. The authors also highlight the clinical implications of undiagnosed cervical infections in pregnant women, particularly their role in increasing the risk of adverse pregnancy outcomes. Although the cervix can use mechanical, chemical, and immunological barriers to protect itself from insults, polymicrobial infections may evade these protective mechanisms. This emphasizes the importance of infection prevention (through interventions such as education and vaccination), early screening and disease management.

Focusing on viral infections, Ikumi and Matjila further review evidence on the role of the placenta in spontaneous PTB and how maternal HIV infection and/or antiretroviral treatment (ART) may modify this risk. The authors review evidence describing the increased risk of PTB observed among pregnant women living with HIV (WLHIV). Although the placenta has immunological mechanisms that protect against infection, pathogens such as HIV can still penetrate these barriers. Maternal HIV infection in turn is associated with morphological and immunological changes, including placental insufficiency and inflammation, that increase the risk of PTB. Although ART in pregnancy improves maternal health and prevents HIV transmission to the fetus, some data have shown that it is associated with an increased PTB risk that varies by ART regimen and timing of initiation.

Pre-eclampsia is another condition that increases the risk of PTB through a complex network of mechanisms that are still being elucidated. Current data suggest that the maternal microbiome may play a role in the pathogenesis of pre-eclampsia. The study by Geldenhuys et al. provides novel data on the diversity of the gut, vaginal and oral microbiome in a hospital-based cohort of pregnant South African women with and without pre-eclampsia. This data also showed that pre-eclampsia was associated with higher diversity in the vaginal microbiome and reduction in the health-promoting *Lactobacillus* species. However, the overall microbial composition did not differ between pre-eclampsia and normotensive women.

## Interventions

Further research is required to understand the pathophysiological pathways by which infections and other conditions in pregnancy contribute to PTB risk. Insights from this research should enhance the development of appropriate evidence-based interventions. These could be routine or targeted interventions that are primarily aimed at preventing the incidence of PTB (5). If PTB cannot be prevented, interventions that improve the survival and quality of life of premature infants are needed. In some settings, effective interventions, such as antenatal corticosteroids and delayed cord clamping, are not readily available and accessible even though they are recommended by the WHO (5). The study by Lategan et al. describes some of these intervention gaps in a resource-limited setting. More specifically, the authors describe the respiratory support needs and outcomes of a prospective cohort of 522 infants weighing <1,801 g that were admitted to two hospital neonatal units in South Africa. They report a mortality rate of 14.1% in their study. While some of these deaths were unavoidable, such as those that occurred among infants with severe congenital malformations, others could have been prevented. Using modelled data, the authors show that the administration of continuous positive airway pressure could cause the greatest reduction in mortality, followed by surfactant replacement therapy and then invasive mechanical ventilation. The absence of all three procedures would increase the mortality rate to 42.1%. These data highlight the importance of drastically increasing the accessibility and coverage of these and other life-saving interventions so that more avoidable deaths can be prevented (6).

Additional studies are required to improve the measurement of PTB at individual and population levels. The antecedents, mediators, causative agents and mechanisms that culminate in PTB also require more research. The study by Malaba et al. highlights some key methodological considerations for future PTB research. These include addressing the measurement and selection biases that often limit the extent to which insights can be drawn from observational study data. The authors also discuss issues related to gestational age data availability and quality at a population-level and how strengthening routine civil registration systems and improving the accessibility of clinical tests such as ultrasounds could bridge this information gap. Collective intersectoral action that considers the economy, education, equity and human rights (2) is required to achieve this goal. Such action should facilitate the achievement of the broader goals of improving the survival, health, and well-being of mothers, newborns, and children (6).
